# How do Australian podiatrists manage patients with diabetes? The Australian diabetic foot management survey

**DOI:** 10.1186/s13047-015-0072-y

**Published:** 2015-04-18

**Authors:** Thomas R Quinton, Peter A Lazzarini, Frances M Boyle, Anthony W Russell, David G Armstrong

**Affiliations:** Department of Prosthetics, Orthotics, & Podiatry, Princess Alexandra Hospital, Ipswich Road Woolloongabba, QLD 4102 Brisbane, Australia; School of Population Health, The University of Queensland, Brisbane, Australia; Allied Health Research Collaborative, Metro North Hospital & Health Service, Queensland Health, Brisbane, Australia; School of Clinical Sciences, Queensland University of Technology, Brisbane, Australia; Department of Diabetes & Endocrinology, Princess Alexandra Hospital, Brisbane, Australia; School of Medicine, The University of Queensland, Brisbane, Australia; Department of Surgery, Southern Arizona Limb Salvage Alliance (SALSA), University of Arizona College of Medicine, Tucson, USA

**Keywords:** Diabetes, Foot, Ulcer, Australia, Survey

## Abstract

**Background:**

Diabetic foot complications are the leading cause of lower extremity amputation and diabetes-related hospitalisation in Australia. Studies demonstrate significant reductions in amputations and hospitalisation when health professionals implement best practice management. Whilst other nations have surveyed health professionals on specific diabetic foot management, to the best of the authors’ knowledge this appears not to have occurred in Australia. The primary aim of this study was to examine Australian podiatrists’ diabetic foot management compared with best practice recommendations by the Australian National Health Medical Research Council.

**Methods:**

A 36-item Australian Diabetic Foot Management survey, employing seven-point Likert scales (0 = Never; 7 = Always) to measure multiple aspects of best practice diabetic foot management was developed. The survey was briefly tested for face and content validity. The survey was electronically distributed to Australian podiatrists via professional associations. Demographics including sex, years treating patients with diabetes, employment-sector and patient numbers were also collected. Chi-squared and Mann Whitney U tests were used to test differences between sub-groups.

**Results:**

Three hundred and eleven podiatrists responded; 222 (71%) were female, 158 (51%) from the public sector and 11–15 years median experience. Participants reported treating a median of 21–30 diabetes patients each week, including 1–5 with foot ulcers. Overall, participants registered median scores of at least “very often” (>6) in their use of most items covering best practice diabetic foot management. Notable exceptions were: “never” (1 (1 – 3)) using total contact casting, “sometimes” (4 (2 – 5)) performing an ankle brachial index, “sometimes” (4 (1 – 6)) using University of Texas Wound Classification System, and “sometimes” (4 (3 – 6) referring to specialist multi-disciplinary foot teams. Public sector podiatrists reported higher use or access on all those items compared to private sector podiatrists (p < 0.01).

**Conclusions:**

This study provides the first baseline information on Australian podiatrists’ adherence to best practice diabetic foot guidelines. It appears podiatrists manage large caseloads of people with diabetes and are generally implementing best practice guidelines recommendations with some notable exceptions. Further studies are required to identify barriers to implementing these recommendations to ensure all Australians with diabetes have access to best practice care to prevent amputations.

## Background

Diabetic foot complications are a major public health problem, causing significant morbidity, mortality and burden on the broader health system [1-4]. In Australia diabetic foot complications are a leading cause of hospital admission for people with diabetes [1,4] and the most common cause of lower limb amputation [5]. The average hospital stays for diabetic foot ulcer and amputation admissions are 12 and 26 days respectively [1,4]. Diabetic foot complications also account for 8% of total deaths secondary to diabetes [1,4]. As Australia has a rapidly increasing diabetes prevalence (currently 7%), with 19.6% of those at risk of foot ulceration, the growing national diabetic foot burden requires equally considerable national evidence based management interventions to combat this significant growing burden [2,4].

Despite both the detrimental and costly effects of diabetic foot complications, effective treatments and preventions are possible [4]. Appropriate care has been shown to significantly reduce diabetes-related amputation rates [6-9]. Internationally a significant effort has been made to reduce diabetic foot complications and a large body of research now exists [10-13]. In Australia health care service providers have made substantial recent investments to try and improve care and patient outcomes for people with diabetic foot complications [4,10,12]. Best practice guidelines based on systematic reviews of the literature for the prevention and treatment of diabetic foot complications are now widely available [4,11-13].

Despite the availability of diabetic foot guidelines and the evidence for their use in improving patient outcomes, the recommendations found within these guidelines are not always adhered to in clinical practice [14-18]. Studies performed in the US report low compliance with certain guideline recommendations in diabetic foot care [14,15]. One large study of US podiatrists found a very low rate of use of the gold standard offloading treatment of total contact casting, but it did not report compliance with any other best practice guideline recommendations [15]. The elements that influence the use of guidelines in clinical practice are complex and multifaceted including individual clinician factors, broader system factors, and guideline specific factors [17,19,20].

Podiatrists play a central role in providing care for diabetic foot complications [21-23]. Most of the recommendations outlined in Australian National Evidence-Based Guideline on the *Prevention, Identification and Management of Foot Complications in Diabetes* apply directly to care provided by podiatrists [4]. However, there is limited literature examining how podiatrists’ clinical practice reflects evidence based practice in Australia [10,16]. One study surveying podiatry centres in Victoria reported over 60% of centres broadly used clinical guidelines when managing patients with diabetic foot disorders but did not provide further detail about compliance with specific guideline recommendations [16]. It has also been identified that the Australian Government’s Medical Benefits Schedule (MBS) does not attribute funding to many of the key evidence based recommendations outlined in the Australian guidelines, such as total contact casting or other devices rendered irremovable [24]. This shortfall in Australian Government MBS rebates for patients needing evidence based diabetic foot care in the private sector is generally supplemented via State Government funding in the public sector, therefore potentially providing public sector podiatrists greater access to evidence based diabetic foot resources than their private sector colleagues [24]. To the best of the authors’ knowledge, no studies have examined how diabetic foot care guideline recommendations are being utilised in Australia and are they utilised differently in different sectors. The aim of this study is to investigate the extent to which podiatrists in Australia are meeting current best practice guideline recommendations for the management of diabetic foot complications and to identify any differences between private and public podiatrists.

## Methods

This cross-sectional study of Australian podiatrists was conducted using an online survey between May and June 2013. The school level ethical review committee at the School of Population Health, University of Queensland approved the study.

### Setting and participants

Eligible participants for the survey were podiatrists registered, and residing, in Australia during the May-June 2013 period of the survey (n = 3746) [25].

### Procedure

Convenience and snowball sampling techniques were used to distribute the online survey. Invitations to participate in the online survey were sent via email to members of each member state association of the Australian Podiatry Council. Email invitations were also distributed via the Queensland Department of Health Podiatry Network. The online survey was promoted during presentations at the Sydney Diabetic Foot Conference (May 2013) and the Australian Podiatry Conference (June 2013). The online survey remained open for a period of four weeks. Reminder emails were sent, via the same organisations, two and three weeks after the initial invitation. All participants were advised that the online survey was voluntary and anonymous.

### Survey instrument

To the best of the authors’ knowledge there is no valid and reliable survey tool to assess podiatrists’ best practice management of diabetic foot complications. For this study, we adapted, with permission, a diabetic ulcer management survey developed to survey podiatrists in the US [15]. The Australian Government National Health and Medical Research Council national evidence-based guideline *Prevention, identification and management of foot complications in diabetes* [4] summary recommendations were then directly used to create a 36-item questionnaire covering five content domains: demographics; years diabetic foot experience and caseload; assessment and prevention of diabetic foot complications; assessment and management of diabetic foot ulceration; access and referral to expert multi-disciplinary teams. Each individual recommendation from the national guideline’s summary recommendations section was translated into question format [4]. Seven-point Likert scales (0 = Never; 7 = Always) were created to measure adherence to each individual best practice diabetic foot summary recommendations. The Likert scale was further defined in terms of percentage of patients each week receiving the item of care by the respondent podiatrist: “1 = never (0%)”, “2 = very rarely (1 – 20%)”, “3 = rarely (21 – 40%)”, “4 = sometimes (41 – 60%), “5 = often (61 – 80%)”, “6 = very often (81 – 99%), and, “7 = always (100%)”. The online software program *SurveyMonkey* was used to host the survey [26].

To ensure the survey had suitable face and content validity it was sent to recognised national experts in diabetic foot care (n = 3) for review and then piloted on a small number of general podiatrists (n = 7) for feedback on practicality, face and content validity. Minor modifications were made to address the feedback of the expert and general podiatrists. Any disputes on changes were agreed by the consensus of the authors.

### Data analysis

Data from the survey were analysed using SPSS 19.0 for Windows (SPSS Inc., Chicago, IL, USA). The internal consistency of the survey itself was tested using Cronbach’s alpha for the clinical items [27]. Descriptive statistics were used to display variable data using numbers and proportions for categorical data unless otherwise indicated [27]. Chi-squared and Mann Whitney U tests were used to test any differences between sub-groups [27]. A minimum significance level of *P* < 0.05 was used throughout .

## Results

There were a total of 311 respondents to the survey (8.3% of all registered Australian podiatrists). The Cronbach’s alpha score for the 37-item survey was 0.876 and categorised as having “good” internal consistency [27]. Table 1 displays the general demographic characteristics of respondents. Table 2 reports the years of diabetes experience and average caseload of respondents. These results indicate respondents treat an average of 21–30 patients with diabetes each week, including an average of 1 – 5 patients with a foot ulcer. Public podiatrists report treating more foot ulcer patients (6 – 10) than private podiatrists (1 – 5) (*p* < 0.001).

**Table 1:** **Demographic details of respondents (number (%) unless otherwise stated)**

**Gender**	**Total**	**Public**	**Private**	***p*** **Value**
Total	311 (100%)	158 (51%)	153 (49%)	
Male	89 (29%)	44 (49%)	45 (51%)	0.760
Female	222 (71%)	114 (51%)	108 (49%)	0.760
**State**				
Qld	121 (39%)	48 (40%)	73 (60%)	
Vic	93 (30%)	53 (57%)	40 (43%)	
NSW	48 (15%)	18 (38%)	30 (62%)	
SA	18 (6%)	12 (67%)	6 (33%)	
WA	18 (6%)	16 (89%)	2 (11%)	
Tas	11 (4%)	10 (91%)	1 (9%)	
NT	2 (1%)	1 (50%)	1 (50%)	
**Region**				
Metropolitan	188 (60%)	99 (53%)	89 (47%)	0.418
Regional and Rural	123 (40%)	59 (48%)	64 (52%)	0.418

**Table 2:** **Diabetes experience and current caseload**

	**Total**	**Public**	**Private**	***p*** **Value**
	**M (IQR)**	**M (IQR)**	**M (IQR)**	
Number of patients with diabetes treated in an average week	21-30	21-30	21-30	0.042
	(11–20 - 31–40)	(11–20 - 31–40)	(11–20 - 31–40)	
Number of patients with diabetic foot ulcers treated in an average week	1-5	6-10	1-5	<0.001
	(1–5 – 6–10)	(1–5 – 21–30)	(0 – 1–5)	
Years treating patients with diabetes (years)	11-15	6-10	11-15	0.052
	(0–5 – 16–20)	(0–5 – 16–20)	(0–5 – 21–25)	

M, median; IQR, interquartile range.

Tables 3, 4 and 5 display the median (interquartile range) results for items on adherence to recommendations found within best practice guidelines. Table 3 demonstrates respondents generally utilise best practice for the assessment and prevention of diabetic foot complications. Respondents registered median scores of at least “very often” for all 13 items in this section, except performing an ankle brachial index (ABI) or toe pressure assessment only “sometimes” (4 (2–5)). Again public podiatrists reported utilising ABI or toe pressure assessments more often than private colleagues (*p* < 0.001).

**Table 3:** **Assessment and prevention of diabetic foot complications (Pre-diabetic foot ulceration)**

	**Total**	**Public**	**Private**	***p*** **Value**
	**M (IQR)**	**M (IQR)**	**M (IQR)**	
Assess for risk of developing foot complications?	7 (6 – 7)	7 (6 – 7)	7 (6 – 7)	0.111
Inquire about previous foot ulcers and amputations?	7 (6 – 7)	7 (6 – 7)	7 (5 – 7)	0.001
Visually inspect feet for structural abnormalities?	7 (7 – 7)	7 (7 – 7)	7 (6 – 7)	0.253
Visually inspect feet for wounds?	7 (7 – 7)	7 (7 – 7)	7 (7 – 7)	0.165
Assess for neuropathy?	7 (6 – 7)	7 (6 – 7)	7 (6 – 7)	0.414
Assess for neuropathy using a 10 g monofilament?	7 (6 – 7)	7 (6 – 7)	7 (6 – 7)	0.122
Palpate their foot pulses?	7 (6 – 7)	7 (6 – 7)	7 (6 – 7)	0.214
Perform an Ankle Brachial Index (ABI) or Toe Pressure assessment?	4 (2 – 5)	4 (3 – 6)	3 (1 – 4)	<0.001
Classify their risk of developing foot complications?	7 (6 – 7)	7 (6 – 7)	7 (5 – 7)	0.149
Provide foot care education to prevent foot complications?	7 (6 – 7)	7 (6 – 7)	7 (6 – 7)	0.709
Provide or recommend footwear to prevent foot complications?	6 (6–7)	6 (6 – 7)	6 (6 – 7)	0.927
Recommend a review assessment annually for low risk patients?	7 (6 – 7)	7 (6 – 7)	7 (6 – 7)	0.721
Recommend a review examination within 6 months for patients with foot risk factors?	7 (6 – 7)	7 (6 – 7)	7 (6 – 7)	0.991

M, median; IQR, interquartile range.

1 = never (0%), 2 = very rarely (1 – 20%), 3 = rarely (21 – 40%), 4 = sometimes (41 – 60%), 5 = often (61 – 80%), 6 = very often (81 – 99%), and, 7 = always (100%).

**Table 4:** **Assessment and management of diabetic foot ulceration**

	**Total**	**Public**	**Private**	***p*** **Value**
	**M (IQR)**	**M (IQR)**	**M (IQR)**	
Believe foot ulcers to be serious requiring immediate management?	6 (5 – 7)	7 (6 – 7)	6.5 (5 – 7)	<0.001
Grade foot ulcer severity based on depth, infection status and peripheral arterial disease status?	6 (4 – 7)	7 (6 – 7)	6 (4 – 7)	<0.001
Grade foot ulcer severity according to the University of Texas Wound Classification System?	4 (1 – 6)	6 (4 – 7)	2 (1 – 4)	<0.001
Perform sharp debridement of non-ischaemic ulcers?	7 (6 – 7)	7 (6 – 7)	6 (5.5 – 7)	0.022
Use topical hydrogel dressings for autolytic debridement of non-ischaemic ulcers?	4 (2 – 5)	4 (3 – 5)	4 (2 – 5)	0.395
Use wound dressings that create a moist wound environment for non-ischaemic ulcers?	6 (5 – 7)	6 (6 – 7)	6 (4 – 7)	< 0.001
Use wound dressings that maintain a dry wound environment for ischaemic ulcers?	5 (3 – 6)	6 (5 – 7)	5 (3 – 6)	< 0.001
Believe that offloading in order to reduce pressure at the ulcer site is necessary to aid healing?	7 (6.5 – 7)	7 (7 – 7)	7 (6 – 7)	0.177
Use total contact casting?	1 (1 – 3)	3 (1 – 4)	1 (1 – 2.5)	< 0.001
Use a removable cast walker rendered irremovable or instant total contact cast?	2 (1 – 4)	4 (2 – 5)	2 (1 – 4)	< 0.001
Use a removable offloading device (for example orthoses, felt, shoe modifications)?	6 (5 – 7)	6 (5 – 6)	6 (5 – 6)	0.781

M, median; IQR, interquartile range.

1 = never (0%), 2 = very rarely (1 – 20%), 3 = rarely (21 – 40%), 4 = sometimes (41 – 60%), 5 = often (61 – 80%), 6 = very often (81 – 99%), and, 7 = always (100%).

**Table 5:** **Access and referral to specialist multi-disciplinary teams**

	**Total**	**Public**	**Private**	***p*** **Value**
	**M (IQR)**	**M (IQR)**		
Do you have access to a specialist multi-d foot team?	212/287 (74%)	120/147 (82%)	92/140 (66%)	0.002
How often did you refer diabetic foot ulceration?	4 (3 – 6)	5 (4 – 6)	4 (3 – 6)	< 0.001
How often did you refer deep foot ulceration (probing to tendon, joint or bone)?	7 (4 – 7)	6 (6 – 7)	7 (2.5 – 7)	0.144
How often did you refer ulcers not reducing in size after 4 weeks?	5 (4 – 7)	5 (4 – 7)	5.5 (3.5 – 7)	0.124
How often did you refer ulcers in patients with absent foot pulses?	6 (4 – 7)	6 (4 – 7)	6 (3.5 – 7)	0.394
How often did you refer ulcers with ascending cellulitis?	7 (5 – 7)	7 (6 – 7)	7 (4 – 7)	0.359
How often did you refer suspected Charcot’s neuroarthropathy?	7 (5 – 7)	7 (6 – 7)	7 (2.5 – 7)	0.688
Do you have access to an expert foot care consultation via telehealth?	31/283 (11%)	29/145 (20%)	2/138 (1%)	< 0.001

M, median; IQR, interquartile range.

1 = never (0%), 2 = very rarely (1 – 20%), 3 = rarely (21 – 40%), 4 = sometimes (41 – 60%), 5 = often (61 – 80%), 6 = very often (81 – 99%), and, 7 = always (100%).

Table 4 reports podiatrists at least “often” utilise best practice for six of the ten diabetic foot ulceration assessment and management items. The other four items scored “sometimes” or less often, including grading ulcers according to the validated University of Texas Wound Classification System, use of topical hydrogel dressings, use of total contact casts and use of removable cast walkers rendered irremovable. Public compared to private podiatrists reported higher rates of use for all these items (*p* < 0.001), except use of topical hydrogel dressings.

Table 5 indicates the majority of respondents (74%) had access to specialist multi-disciplinary teams and at least “often” utilised these teams to refer patients with complex foot ulceration and Charcot’s neuroarthropathy. Public podiatrists reported greater access to these teams (82%) compared to private podiatrists (66%) (*p* < 0.01). Public podiatrists were more likely to refer patients with diabetic foot ulceration to these teams “often” (5 (4–6)) compared to “sometimes” for those in the private sector (4 (3–6)) (*p* <0.001). A small percentage of respondents had access to expert consultation via telehealth (11%); public podiatrists (20%) had greater access than private (1%) (*p* <0.001).

## Discussion

This study reports what, to our knowledge, is the first survey of Australian podiatrists’ practice in relation to diabetic foot best practice guideline recommendations. The survey had good internal consistency and high face and content validity for measuring the diabetic foot management construct. Furthermore, the results are based on what appears to be the largest sample of any Australian survey of podiatrists. Overall, the findings suggest Australian podiatrists treat a significant number of people with diabetes and generally adhere to best practice guidelines with some key exceptions. The most notable exception is a very low rate of irremovable offloading device use, which aligns with the results reported in similar international studies [14,15].

In terms of clinical practice, the suggestion is that Australian podiatrists “very often” adhere to the vast majority of best practice guideline recommendations in relation to assessment, examination, risk classification and review periods when managing patients with diabetes. Performing a comprehensive examination of the foot, including neurovascular and musculoskeletal assessments, is core general podiatry practice recommended by the Australian and New Zealand Podiatry Accreditation Council [28], thus, a high adherence rate to the diabetes guidelines for these items that relate is understandable. Performing an ABI or toe pressure was the only item to score below “very often” and aligned with a similar finding in a recent study of Western Australian podiatrists that reported 63% of podiatrists use the ABI [29]. These findings may also be expected as guideline recommendations typically suggest using an ABI or other non-invasive vascular assessment when pedal pulses are not readily palpable [4]. With peripheral arterial disease rates reported in approximately 15 – 25% of Australians with diabetes [2,30] it is not unexpected that podiatrists only “sometimes” use non-invasive vascular assessments to detect peripheral arterial disease.

Furthermore, guideline recommendations were “very often” adhered to for most diabetic foot ulcer assessment and management items However, notable exceptions were the “never” and “very rare” use of total contact casts and irremovable cast walkers respectively and the “sometimes” use of the validated University of Texas Wound Classification System [31] and topical hydrogels. The recommendation of irremovable offloading (including total contact casting and irremovable cast walkers) device use has the highest level of evidence of all Australian diabetic foot guideline recommendations to optimise foot ulcer healing [4,32]. In contrast, evidence to support the use of removable offloading to heal foot ulcers has amongst the lowest level of evidence reported in diabetic foot management guidelines [4,32]. Our findings suggest that this evidence is not reflected in clinical practice. Contrary to recommendations, podiatrists report using removable offloading devices (including orthoses and felt padding) “very often” when treating their patients with diabetic foot ulcers but use irremovable cast walkers “very rarely” and total contact casts “never”. This finding has also been reported in other international studies indicating that further work is urgently required to encourage the routine use of irremovable offloading devices in clinical practice for the management of patients with diabetic foot ulceration [14,15].

To the best of the authors’ knowledge there is little or no research on clinician compliance with the use of topical hydrogels or the University of Texas Wound Classification System in clinical practice. Although topical hydrogels have a relatively high level of evidence it is primarily for autolytic debridement of wounds rather than as a wound dressing [4]. Thus, again it is understandable that podiatrists who regularly use what has been reported to be a much more efficient debridement modality with sharps debridement [4] do not routinely use topical hydrogels. Furthermore, whilst the University of Texas Wound Classification System was only “sometimes” used by podiatrists to classify the complexity of foot ulcers, they did report “very often” using some form of foot ulcer classification system that still included the necessary best practice assessment components of depth, infection and peripheral arterial disease to categorise foot ulcer severity [4]. This suggests the vast majority of patients seeing a podiatrist for foot ulcer management are receiving best practice guideline recommendations on foot ulcer assessment. Reassuringly, public podiatrists who report treating a much higher volume of patients with foot ulcers report using the validated research quality University of Texas system [31] “very often” in their clinical practice.

The majority of podiatrists indicated they had referral access to expert multidisciplinary diabetic foot teams consistent with guideline recommendations [4]. However, it must be noted that more than one third of private podiatrists did not have access to these teams when the acuity of their patients need it. Whilst this access appears to be increasing compared to other Australian reports [16,33] there still appears to be a significant number of podiatrists, and their patients, that do not have the necessary access to these limb-saving services. Multi-disciplinary teams are vital for best practice diabetic foot ulcer treatment and amputation prevention [4,6-9]. This is an important finding as the exact number and location of expert multidisciplinary foot care teams in Australia is currently not clear [33]. However, some studies indicate that there are grossly insufficient numbers of multidisciplinary diabetic foot care teams in Australia and that they are generally located at metropolitan tertiary hospitals [10,33]. Interestingly, in a country as vast as Australia, the podiatrists in this survey reported very low access to these multidisciplinary experts via telehealth. Access to wound care experts via telehealth has high level evidence in Australia to improve access for rural and remote clinicians’ in particular and studies have demonstrated significantly reductions in amputation rates [4,32,34]. It is strongly recommended that strategies to increase access to multi-disciplinary diabetic foot teams, including via in person and telehealth avenues, are optimised to ensure people with diabetes foot ulcers in Australia are receiving best practice guideline recommendations to significantly reduce their chance of amputation [4].

Sector of employment appears to have an effect on adherence to best practice guidelines. For all items reporting a low adherence rate, public-employed podiatrists reported higher adherence rates than private-employed podiatrists and also had greater access to multidisciplinary teams. Chen et al. also found that rates of ABI use were also higher in public sector than private podiatry [29]. There are a variety of hypotheses why this may be the case including that public podiatrists see a larger volume of patients with diabetic foot ulcers in particular, and thus, more inclined to be aware of and use best practice management recommendations. However, a number of papers have reported concern that the current Australian health system’s Medicare funding model for chronic disease in Australia may be inadequate to deal with patients affected by complex chronic disease [24,35-37]. The Medicare model appears to provide some access for patients with non-complex chronic disease, yet is inadequate in providing best practice care for those with more complex chronic conditions such diabetic foot ulcers [24,35-37]. Alternatively, public podiatrists are supported by government funding at a state level to implement best practice care and this has been shown to improve clinical practice adherence to best practice guidelines [10] and in turn significantly reduces diabetic foot hospitalisation and amputation [38,39].

Interestingly, the findings from this study suggest that Australian podiatrists manage significant caseloads of patients with diabetes each week (median 21 – 30 patients per week). This median range extrapolated to all registered Australian podiatrists equates to approximately 75,000 – 110,000 diabetes-related podiatry consultations occurring weekly in Australia. Similarly, podiatrists report managing 1 – 5 diabetes patients with foot ulcers on average each week, equating to approximately 3,700 – 18,000 podiatry consultations each week. Whilst these figures are encouraging in terms of capacity to treat Australia’s burgeoning diabetes foot ulcer population, it still appears significant further capacity is required to manage the minimum estimated 20,000 Australians (2% of Australia’s diabetes population) requiring multi-disciplinary management of their diabetic foot ulcer each week [24] to meet best practice guideline recommendations [4].

### Limitations

Whilst this study reports some interesting findings they should be read cognisant of a number of limitations. Firstly, the sampling technique used by this study is likely to have caused sampling bias. As the study was voluntary, entitled ‘diabetic foot management’ and promoted at conference sessions discussing diabetic foot complications it could be suggested that the majority of podiatrists more likely to complete such a survey are those interested in diabetic foot management, and thus, more likely to adhere to guideline recommendations. An example of this is the over representation of public sector podiatrists completing this survey; 50% of respondents for this survey indicated they were publicly employed, whilst only 20% of registered Australian podiatrists are publicly employed [25,40]. Secondly, using a non-validated survey tool decreases the reliability and external validity of our results. However, the survey was based on a similar US survey [15] and with direct questions and phrasing from specific Australian diabetic foot guideline recommendations [4], the questionnaire appears to have suitable content and face validity as indicated by a small sample of expert and general podiatrists and had good internal. Thirdly, the method of survey distribution did not allow for the determination of an exact denominator population of podiatrists invited, and thus, a response rate could not be calculated. The authors anticipate the survey invitation reached a minimum of 2,000 podiatrists who were members of the Australian Podiatry Associations but would not have reached all registered podiatrists (n = 3746) [25]. Thus, the estimated response rate would range between 8 – 16% and acknowledged as a low response rate. However, the high female proportions (70%) and median years of experience (11–15 years) are representative of the Australian registered podiatry workforce [40]. Furthermore, 311 respondent podiatrists appear to make this the largest survey of Australian podiatrists published to date. Lastly, the authors are cognisant of potential response bias by participants completing the survey as they believe the investigators would like it to be completed. The authors attempted to address this in the participant information prior to survey completion which advised participants that the survey results were anonymous and “this survey is not a test of your knowledge. Answers that reflect the way you actually practice will give the most meaningful results.”

Our survey did not gather information on why guideline recommendations were or were not followed. Fife et al. found that there are a number of specific barriers to the utilisation of best practice in diabetic foot ulcer care. For example, the TCC is time consuming, difficult to apply, and there are risks involved with its use. [14]. Further research examining these and other barriers and enablers at both the individual and system level are strongly recommended if we are to ensure people with diabetes foot complications have access to best practice care in Australia. This study suggests that public sector podiatrists may be implementing best practice recommendations in diabetic foot care significantly more often than private sector podiatrists. Considering the increasing prevalence of diabetes and the limited public health budget it is important the reasons for these differences are identified and addressed where possible [41].

## Conclusion

This study provides useful initial information on Australian podiatrists’ clinical management of patients with diabetic foot disease. It appears podiatrists are generally implementing best practice guidelines in the management of diabetic foot disease with some key exceptions and these exceptions require further investigation. Identifying the barriers and enablers around these guideline recommendations would help to inform policy and funding decisions in this important area and hopefully lead to improved diabetes health outcomes, improved amputation rates and significantly reduced costs on the broader Australian health system.

## References

[1] Australian Institute of Health & Welfare (AIHW) (2008). Diabetes: Australian facts 2008.

[2] Tapp RJ, Shaw JE, Courten MPD, Dunstan DW, Welborn TA, Zimmet PZ (2003). Foot complications in type 2 diabetes: an Australian population–based study. Diabet Med.

[3] Armstrong DG, Wrobel J, Robbins JM (2007). Guest Editorial: are diabetes related wounds and amputations worse than cancer?. Int Wound J.

[4] National Health & Medical Research Council (NHMRC) Guidelines (2011). National evidence-based guideline on prevention, identification and management of foot complications in diabetes (Part of the guidelines on management of type 2 diabetes).

[5] Lazzarini PA, O’Rourke SR, Russell AW, Clark D, Kuys SS (2012). What are the key conditions associated with lower limb amputations in a major Australian teaching hospital?. J Foot Ankle Res.

[6] Krishnan S, Nash F, Baker N, Fowler D, Rayman G (2008). Reduction in diabetic amputations over 11 years in a defined U.K. population: benefits of multidisciplinary team work and continuous prospective audit. Diabetes Care.

[7] Canavan RJ, Unwin NC, Kelly WF, Connolly VM (2008). Diabetes and nondiabetes related lower extremity amputation incidence before and after the introduction of better organized diabetes foot care: continuous longitudinal monitoring using a standard method. Diabetes Care.

[8] Aydin K, Isildak M, Karakaya J, Gürlek A (2010). Change in amputation predictors in diabetic foot disease: effect of multidisciplinary approach. Endocrine.

[9] van Houtum W, Rauwerda J, Ruwaard D, Schapper N, Bakker K (2004). Reduction in diabetes related lower extremity amputations in the Netherlands: 1991–2000. Diabetes Care.

[10] Lazzarini PA, ORourke SR, Russell AW, Derhy PH, Kamp MC (2012). Standardising practices improves clinical diabetic foot management: the Queensland Diabetic Foot Innovation Project, 2006–09. Aust Health Rev.

[11] National Institute for Health and Clinical Excellence (NICE) (2011). Diabetic foot problems. Patient management of diabetic foot problems.

[12] Department of Health, Western Australia (2010). High risk foot model of care.

[13] Apelqvist J, Bakker K, van Houtum WH, Schaper NC (2008). Practical guidelines on the management and prevention of the diabetic foot: based upon the international consensus on the diabetic foot. Diabetes Metab Res Rev.

[14] Fife CE, Carter MJ, Walker D (2010). Why is it so hard to do the right thing in wound care?. Wound Repair Regen.

[15] Wu SC, Jensen JL, Weber AK, Robinson DE, Armstrong DG (2008). Use of pressure offloading devices in diabetic foot ulcers: do we practice what we preach?. Diabetes Care.

[16] Bergin SM, Brand CA, Colman PG, Campbell DA (2009). An evaluation of community-based resources for management of diabetes related foot disorders in an Australian population. Aust Health Rev.

[17] Prompers L, Huijberts M, Apelqvist J, Jude E, Piaggesi A, Bakker K (2008). Delivery of care to diabetic patients with foot ulcers in daily practice: results of the Eurodiale Study, a prospective cohort study. Diabet Med.

[18] Lawrence SM, Wraight PR, Campbell DA, Colman PG (2004). Assessment and management of acute diabetes related foot complications: room for improvement. Inter Med J.

[19] Lizarondo L, Grimmer-Somers K, Kumar S (2011). A systematic review of the individual determinants of research evidence use in allied health. J Multidiscip Healthc.

[20] Lugtenberg M, Zegers-van Schaick JM, Westert GP, Burgers JS (2009). Why don’t physicians adhere to guideline recommendations in practice? An analysis of barriers among Dutch general practitioners. Implement Sci.

[21] Boulton AJ, Armstrong DG, Albert SF, Frykberg RG, Hellman R, Kirkman MS (2008). Comprehensive foot examination and risk assessment a report of the task force of the foot care interest group of the American diabetes association, with endorsement by the American association of clinical endocrinologists. Diabetes Care.

[22] Sloan FA, Feinglos MN, Grossman DS (2010). Receipt of care and reduction of lower extremity amputations in a nationally representative sample of US elderly. Health Serv Res.

[23] Skrepnek GH, Mills JL, Armstrong DG. Foot-in-wallet disease: tripped up by ‘cost-saving’ reductions. Diabetes Care. 2014. In Press 10.2337/dc14-0079 25147261

[24] Lazzarini PA, Gurr JM, Rogers JR, Schox A, Bergin SM (2012). Diabetes foot disease: the Cinderella of Australian diabetes management?. J Foot Ankle Res.

[25] Australian Health Practitioner Regulation (2013). Podiatry registrant data: March 2013.

[26] Survey Monkey®. Available: https://www.surveymonkey.com/ Website-[Accessed 03/03/2014]

[27] Kirkwood B, Sterne J (2003). Essential medical statistics: second edition.

[28] Australian and New Zealand Podiatry Accreditation Council (2009). Podiatry competency standards for Australia and New Zealand.

[29] Chen PY, Lawford KM, Shah N, Pham J, Bower VM (2012). Perceptions of the ankle brachial index amongst podiatrists registered in Western Australia. Journal of Foot and Ankle Research.

[30] Lazzarini PA, O’Rourke SR, Russell AW, Derhy PH, Kamp MC, dE MC (2013). Queensland’s high risk foot database: tracking the length and width of Queensland’s foot ulcers. J Foot Ankle Surg.

[31] Armstrong DG, Lavery LA, Harkless LB (1998). Validation of a diabetic wound classification system. The contribution of depth, infection, and ischemia to risk of amputation. Diabetes Care.

[32] Bus SA, Valk GD, van Deursen RW, Armstrong DG, Caravaggi C, Hlavacek P (2008). The effectiveness of footwear and offfloading interventions to prevent and heal foot ulcers and reduce plantar pressurs in diabetes: a systematic review. Diabetes Metab Res Rev.

[33] Lazzarini PA, Clark D, Mann RD, Perry VL, Thomas CJ, Kuys SS (2010). Does the use of store-and-forward telehealth systems improve outcomes for clinicians managing diabetic foot ulcers? A pilot study. Wound Practice & Research.

[34] Santamaria N, Carville K, Ellis I, Prentice J (2004). The effectiveness of digital imaging and remote expert wound consultation on healing rates in chronic lower leg ulcers in the Kimberley region of Western Australia. Primary Intention.

[35] Bergin SM, Alford JB, Allard BP, Gurr JM, Holland EL, Horsley MW (2012). A limb lost every 3 hours: can Australia reduce lower limb amputations in people with diabetes?. Med J Aust.

[36] Short A (2009). Footing the bill: the introduction of Medicare Benefits Schedule rebates for podiatry services in Australia. Journal of Foot and Ankle Research.

[37] Foster MM, Mitchell G (2008). Does enhanced primary care enhance primary care? Policy-induced dilemma’s for allied health professionals. Med J Aust.

[38] Lazzarini PA, O’Rourke SR, Russell AW, Derhy PH, Kamp MC (2011). Standardising practices improves ambulatory diabetic foot management and reduces amputations: the Queensland Diabetic Foot Innovation Project, 2006–2009. Journal of Foot and Ankle Research.

[39] Lazzarini PA, O’Rourke SR, Russell AW, Derhy PH, Kamp MC (2013). Reduction in the incidence of diabetes lower extremity amputations in Queensland: 2005–2010. Journal of Foot and Ankle Research.

[40] Health Workforce Australia (HWA) (2014). Australia’s health workforce series – podiatrist in focus.

[41] Whiting DR, Guariguata L, Weil C, Shaw J (2011). IDF diabetes atlas: global estimates of the prevalence of diabetes for 2011 and 2030. Diab Res Clin Prac.

